# 30例双原发肺癌的临床及预后因素分析

**DOI:** 10.3779/j.issn.1009-3419.2017.10.02

**Published:** 2017-10-20

**Authors:** 子奇 王, 晶晶 侯, 慧娟 王, 国伟 张, 智勇 马

**Affiliations:** 1 450008 郑州，郑州大学附属肿瘤医院呼吸内科 Department of Respiratory Medicine, the Afliated Cancer Hospital of Zhengzhou University, Zhengzhou 450008, China; 2 454150 焦作，焦作市第二人民医院肿瘤内科一病区 Department of Internal Medicine-Oncology, Ward One, the Second People's Hospital of Jiaozuo City, Jiaozuo 454150, China

**Keywords:** 肺肿瘤, 多原发肺癌, 双原发肺癌, 临床特征, 治疗, 预后, Lung neoplasms, Multiple primary lung cancer, Double Primary Lung Cancer, Clinical features, Treatment, Prognosis

## Abstract

**背景与目的:**

多原发肺癌（multiple primary lung cancer, MPLC）是一种临床中较为少见的肺癌类型，双原发肺癌（double primary lung cancer, DPLC）是其中最常见的一种，近年来由于诊疗手段的进步检出率逐渐升高。本研究总结分析了30例DPLC患者的临床资料，以期为DPLC的诊疗提供理论依据。

**方法:**

回顾郑州大学附属肿瘤医院2010年1月-2015年12月收治的30例DPLC患者的临床资料，对临床特征及预后相关因素进行分析。

**结果:**

30例中，同时性双原发癌（synchronous DPLC, sDPLC）占3例（3/30, 10%），异时性双原发癌（metachronous DPLC, mDPLC）占27例（27/30, 90.0%）。病灶好发于右肺上叶（20/60, 33.3%），病理类型以腺癌（25/60, 41.7%）为主，病理类型相同者（17/30, 56.7%）多于不同者（13/30, 43.3%），病理类型相同者以腺-腺（10/16, 62.5%）最常见。生存分析显示淋巴结转移（HR=4.349, 95%CI: 1.435-13.178, *P*=0.009）和重度吸烟史（HR=2.996, 95%CI: 1.089-8.240, *P*=0.034）是DPLC的不良预后因素。

**结论:**

DPLC好发于右肺上叶，病理类型以腺癌为主，早期诊断、积极的治疗和严格的戒烟策略有望改善其预后。

## Abstract (en)

**Background and objective:**

Multiple primary lung cancer (MPLC) represents a rare variety of pulmonary malignant tumor while mainly consist of double primary lung cancer (DPLC), but the development of diagnostic techniques promotes the rate diagnosis of it in recent years. Here we summarize and anylasis the data of 30 cases with DPLC in expectation of improving congnitive of MPLC.

**Methods:**

We retrospectly studied 30 MPLC patients who diagnosed from January 2010 to December 2015 in Affiliated Cancer Hospital of Zhengzhou University. The clinical characteristics, diagnosis, therapeutic methods and prognosis were analyzed.

**Results:**

Among 30 patients enrolled, metachronous DPLC (27/30, 90%) is far more common than synchronous DPLC. The majority of the lesions located at right upper lobe (20/60, 33.3%), and adenocarcinoma (25/60, 41.7%) turns out to be the major pathological patter. Patients with same pathological pattern of two lesions of DPLC (17/30, 56.7%) were slightly more than those with different pattern (13/30, 43.3%) and adeno-adeno was the most common pattern in the former ones. *Cox* regression anylasis shows that lymphatic metastasis (HR=4.349, 95%CI: 1.435-13.178, *P*=0.009) and experience of heavy smoking (HR=2.996, 95%CI: 1.089-8.240, *P*=0.034) were associate with worse prognosis.

**Conclusion:**

DPLC are mainly located at right upper lobe, and mostly adenocarcinomas. Earlier diagnosis, active therapeutic approaches and a strict smoking cessation strategy may improve the prognosis of MPLC.

肺癌在各种恶性肿瘤中的发病率和死亡率均居第一位^[1]^，多原发肺癌（multiple primary lung cancer, MPLC）是其中较为少见的一种类型，而双原发肺癌（double primary lung cancer, DPLC）为其中最多见类型^[2, 3]^。DPLC是指在患者肺内同时或先后发生两个原发恶性肿瘤，根据病灶间诊断时间间隔是否大于6个月可以分为同时性双原发癌（synchronous DPLC, sDPLC）和异时性双原发癌（metachronous DPLC, mDPLC）。近年来，随着诊断及治疗手段的进步以及人口老龄化的加剧，DPLC的发病率和检出率逐年升高^[4]^，但是关于DPLC的诊疗及预后尚无定论，本研究回顾性分析30例DPLC患者的资料，分析其病理特征以及预后影响因素，以期为DPLC的诊疗积累经验，进一步加深对DPLC的认识。

## 1 资料与方法

### 1.1 一般资料

收集2010年1月-2015年12月在郑州大学附属肿瘤医院就诊的30例DPLC患者的病历资料，占同期收治肺癌患者的0.26%（30/11, 631），与既往报道^[2-7]^相似。病例的筛选参考Martini和Melamed（M-M）于1975年建立的DPLC诊断标准，包括：sMPLC：①病灶位于不同部位，互相独立；②组织学类型不同；③组织学类型相同时需满足以下条件：位于不同的肺段、肺叶或双侧肺，且起源于不同的原位癌，共同的淋巴引流部位无癌，诊断时无肺外转移。mMPLC：①组织学类型不同；②若组织学类型相同，需满足以下任1条：a.无瘤间隔期≥2年；b.起源于不同的原位癌；c.再发原发癌位于不同肺叶或对侧肺，且共同的淋巴引流部位无癌，确立诊断时无肺外转移。30例共60个病灶，其中39例病灶病理类型的确定基于术后标本，15例基于经皮穿刺肺活检（percutaneous aspiration biopsy of lung, PABL），4例基于经支气管镜肺活检（transbronchiallung biopsy, TBLB），2例基于淋巴结穿刺活检（lymph node biopsy）。

### 1.2 随访方法

随访方法采用门诊或电话随访，随访日期截止至2016年11月31日，总生存期（overall survival, OS）的计算从病理确诊之日开始至死亡当日或者末次随访时间。

### 1.3 分期方法

分期按照肺癌肿瘤-淋巴结-转移（tumor-node-metastasis, TNM）分期（第8版）进行，对各病灶单独进行分期，最终以最高分期为准。

### 1.4 统计学方法

采用SPSS 17.0软件进行统计学分析，率的比较使用卡方检验。采用*Kaplan-Meier*法进行生存分析，并进行*Log-rank*检验，采用*Cox*模型进行多因素回归分析。检验水准*α*=0.05。*P* < 0.05为差异有统计学意义。

## 2 结果

### 2.1 患者及病灶的临床病理特征

2010年1月-2015年12月我院收治MPLC 30例，占同期收治肺癌的0.26%（30/11, 631）。30例患者均为DPLC。患者临床特征详见表 1。患者第一原发癌确诊时中位年龄为60.5岁（33岁-71岁），以男性（26/30, 86.7%）和mDPLC（27/30, 90%）为主，重度吸烟患者（吸烟指数 > 400）占50%（15/30）。mDPLC患者两癌确诊间隔中位时间为66.5个月（9个月-324个月）。第一和第二原发癌的病理类型相同者稍多于不同者（17/30, 56.7%），相同者以腺-腺为主（10/17, 58.8%）。出现淋巴结转移的患者占56.7%（17/30）。sMPLC的临床病理特征如下：男性占66.7%（2/3），重度吸烟者占66.7%（2/3），病理类型相同者占33.3%（1/3），有淋巴结转移者占66.7%（2/3）；mDPLC临床病理特征如下：男性88.9%（24/27），重度吸烟者48.1%（13/27），病理类型相同者59.3%（16/27），有淋巴结转移者59.3%（16/27），卡方检验显示各特征均无差异（*P*=0.283, *P*=0.626, *P*=0.390, *P*=0.804）。3例诊断为sDPLC的患者中，2例患者两次病灶病理类型不同（分别为腺-鳞、腺-小），1例患者两次病灶病理类型相同（腺-腺），但位于不同侧肺（左肺下叶-右肺上叶），两侧病灶均无淋巴结转移（Ⅰa-Ⅰb），两侧病灶均接受了突变阻滞扩增系统（amplification refractory mutation system, ARMS）*EGFR*基因检测，结果显示右肺上叶病灶为EGFR 19外显子Del突变（扩增曲线见图 1A），左肺下叶为*EGFR*野生型（图 1B）。30例患者共有60例病灶，其特征详见表 2。60例病灶中位于右肺上叶者居多（20/60, 33.3%），TNM分期以Ib期居多（25/60, 41.7%），病理类型以腺癌为主（25/60, 41.7%），鳞癌次之。共有55例（5/60, 83.3%）接受了以手术为主的综合治疗。第一原发癌（26/29, 89.7%）和第二原发癌（21/26, 80.8%）的手术方式均以叶切+纵隔淋巴结清扫为主。未接受手术的病灶接受了保守治疗。共有6例病灶接受了*EGFR*基因检测，其中2例检测到*EGFR*突变（1例为21外显子L858R突变，1例为19外显子Del突变）。

**1:** 患者的临床特征 Clinical characteristics of patients

Characteristics	Data
Total	30 (100.0%)
Gender	
Male	26 (86.7%)
Famale	4 (13.3%)
Median age (yr)	60.5
Heavy smoker	
Yes	16 (53.3%)
No	14 (46.7%)
Lymphatic metastasis	
Yes	17 (56.7%)
No	13 (43.3%)
Location	
Ipsilateral but not same lobe	12 (40.0%)
Bilateral	18 (60.0%)
Pathological pattern	
Same	17 (56.7%)
Adeno-adeno	10 (58.8%)
Squa-squa	6 (35.3%)
SCC-SCC	1 (5.6%)
Different	13 (43.3%)
Adeno-squa	4 (30.8%)
Adeno-SCC	1 (7.7%)
Adeno-LCC	1 (7.7%)
Squa-SCC	6 (46.2%)
Squa-LCC	1 (7.7%)
Adeno: adenocarcinoma; Squa: squamous carcinoma; SCC: small cell carcinoma; LCC: large cell carcinoma.	

**1:**
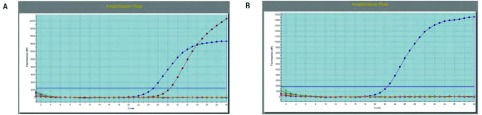
EGFR扩增曲线。A：突变患者扩增曲线；B：野生型患者扩增曲线。 EGFR amplification plots. A: Plots of EGFR+ patient; B: Plots of EGFR-patient.

**2:** 病灶的特征 Characteristics of the lesions

Clinical characteristics	Data
Total	60 (100.0%)
Location	
Right upper lobe	20 (33.3%)
Right middle lobe	4 (6.7%)
Right lower lobe	7 (11.7%)
Right hilus	4 (6.7%)
Right mediastina	1 (1.7%)
Left upper lobe	11 (18.3%)
Left lower lobe	12 (20%)
Left mediastina	1 (1.7%)
Pathological pattern	
Adenocarcinoma	26 (43.3%)
Squamous carcinoma	23 (38.3%)
Small cell carcinoma	9 (15.0%)
Large cell carcinoma	2 (3.3%)
TNM	
Ⅰa	11 (18.3%)
Ⅰb	25 (41.7%)
Ⅱa	6 (10.0%)
Ⅱb	12 (20%)
Ⅲa	1 (1.7%)
Ⅲb	2 (3.3%)
Ⅳ	3 (5.0%)
Surgery	
Yes	55 (91.7%)
FPLC	29 (52.7%)
Lobectomy	26 (89.7%)
Wedge resection	2 (6.9%)
Total pneumonectomy	1 (3.4%)
SPLC	26 (47.3%)
Lobectomy	21 (80.1%)
Wedge resection	4 (15.4%)
Total pneumonectomy	1 (3.8%)
No	5 (8.3%)
FPLC	1 (20.0%)
Chemotherapy	1 (100.0%)
SPLC	4 (80.0%)
Chemotherapy	3 (75.0%)
Concurrent chemo-radiation	1 (25.0%)
FPLC: fast protein liquid chromatography; SPLC: second primary lung cancer; TNM: tumor-node-metastasis.	

### 2.2 预后

30例患者的中位随访时间为70.5个月（17个月-325个月），至2016年11月31日末次随访，共有20例患者死亡，8例患者存活，2例患者失访，失访率6.7%。患者总体中位OS为92.5个月（21个月-325个月），2年生存率90.0%，5年生存率86.7%，若以第二原发癌为起点，则中位OS为19.5个月（1个月-97个月）。通过*Kaplan-Meier*法进行单因素分析结果如表 3所示，其中淋巴结转移（*P*=0.001）（图 2A）和重度吸烟史（*P*=0.007）（图 2B）对于OS的影响具有统计学意义。初次就诊年龄（*P*=0.347）、第一及第二原发癌病理类型是否一致（*P*=0.870）、病灶是否位于同一侧肺部（*P*=0.175）、最高TNM分期（*P*=0.241）对OS的影响无统计学意义，且sDPLC与mDPLC的OS并无统计学差异（*P*=0.436）。*Cox*多因素分析显示重度吸烟史和淋巴结转移是DPLC患者的独立预后因素（表 4）。

**3:** 单因素分析结果 Univariate analysis for survival of 30 cases with MPLC

Variables	*n*	Chi-square	*P*
Age (yr)	11	0.885	0.347
< 60	19		
≥60			
Heavy smoker		7.393	0.007
Yes	16		
No	14		
mMPLC/sMPLC		0.065	0.065
mMPLC	27		
sMPLC	3		
Location		1.838	0.175
Ipsilateral	12		
Bilateral	18		
Lymphatic metastasis		10.154	0.001
Yes	17		
No	13		
TNM		1.377	0.241
Ⅰ-Ⅱ	24		
Ⅲ	3		
Ⅳ	3		
Pathological pattern		1.87	0.127
Same	17		
Different	13		
Interval time（mo）		0.145	1.926
< 24	10		
≥24	20		

**2:**
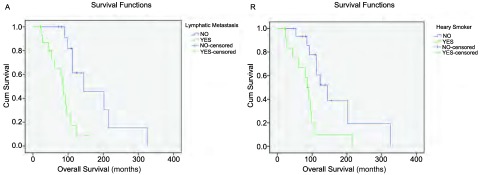
生存曲线。A：有/无淋巴结转移患者的生存曲线；B：有/无重度吸烟史患者的生存曲线。 Survival curve of the patients. A: Survival curve of patients with/without LM; B: Survival curve of patients with/without HS history. LM: lymphatic metastasis; HS: heavy smoker.

**4:** *Cox*回归分析结果 Result of *Cox* regression analysis

Parameters	HR	95%CI	*P*
LM	4.349	1.435-13.178	0.009
HS	2.996	1.089-8.240	0.034

## 3 讨论

1924年Beyreuther^[8]^首次报道了DPLC，此后关于DPLC的报道才开始出现，既往研究所报道的发病率为0.2%-8%不等^[2-7]^，但至今关于DPLC发病率仍无大规模的确切统计报道。Martini和Melamed于1975年提出了DPLC诊断的临床病理学标准，此标准以不同病灶间的发病时间、解剖学位置及病理学类型作为诊断的基础，由于简单方便，可操作性强，广泛被临床医师使用，随后Antakli^[9]^及Rubins等^[10]^相继对Martini-Melamed标准进行了修订，主要的更改是将无瘤间期由2年延长为4年，各个修订版本都强调，相对于判断新发病灶是原发还是转移，更为重要的是判断其是否能接受治愈性的治疗方式。但是仅依靠上述标准诊断多原发病灶依旧存在一定的困难，缺乏分子生物学理论的支撑。进入21世纪后，美国胸科医师联盟（American College of Chest Physicians, ACCP）再次对Martini-Melamed标准进行了修订，引入了分子基因诊断作为标准，提出利用不同的分子遗传学特点对相同病理类型的多发病灶加以鉴别^[11]^。研究者们开始试图利用不同的分子遗传学指标来协助MPLC的诊断，如基因拷贝数^[12, 13]^、杂合性丢失（loss of heterozygosity, LOH）^[14, 15]^、*EGFR*/*Kras*突变状态^[16]^、微卫星标记等位基因变异^[17]^等，但是上述研究的样本量均较小，且实施具有一定的技术难度，尚没有哪种方法得到广泛的认可，但是随着靶向治疗的飞速进展，通过EGFR检测来协助MPLC的诊断对于患者来说似乎是性价比较高的一种选择，有研究者认为仅依靠第一和第二次原发癌*EGFR*突变情况的异同便足以诊断多原发肺癌^[16]^，但是曾有报道^[18]^显示非小细胞肺癌原发灶和转移灶的*EGFR*基因表达存在不一致性。EGFR检测对于MPLC诊断具有一定的价值，但是这种价值的大小还有待进一步的验证。本研究中有1例71岁女性患者同时接受了两次原发灶的EGFR检测，结果一处病灶为*EGFR* 19 Del突变，而另一原发灶为*EGFR*野生型，一定程度上验证了MPLC的诊断。MPLC的诊断需要多学科综合进行，诊断时需要综合考虑组织学类型、遗传学特点、影像学及临床表现等，以期将其与转移病灶准确区分，最大限度降低假阴性率。

本研究显示sDPLC与mDPLC的临床病理特征无明显差异，可能与同时性双原发癌病例数太少有关。DPLC好发于男性，最主要的病理类型为腺癌，病理类型相同者多于不同者，相同者以腺-腺居多，与李营^[3]^、郭海法^[2]^等所报道的相同，而一些早期研究^[4-6]^则显示MPLC好发于男性，病理类型以鳞癌为主，这可能与肺癌近年来的流行病学的改变有关，即肺腺癌发病率逐渐上升，而肺鳞癌则反之^[19, 20]^。本研究的30例患者中，DPLC好发于右肺上叶、同侧多于双侧，这些均与既往的报道相似^[21, 22]^。此外，本研究中入组的患者多数为早期患者（24/30, 80%），这可能与DPLC的诊断标准有关，即在其两次病灶组织学类型相同时，M-M标准中均要求共同的淋巴引流部位无癌，且诊断时无肺外转移方可确立sDPLC的诊断，而这也是两次病灶组织学类型相同时欲诊断mDPLC的条件之一，这就将一部分疾病诊断时的分期限制到了较早的阶段。此外，本研究中的病例绝大多数是mDPLC，而mDPLC的定义中强调两次病灶发现时间必须大于6个月，且M-M标准指出，在组织学类型相同时，无瘤间隔期≥2年也是诊断mDPLC的标准之一，而这些便对患者在首发癌接受治疗后的生存时间提出了一些要求，由于晚期肺癌患者预后明显差于早期接受手术患者，首发癌处于早期的患者便更有可能出现第二次原发癌，此外，患者在罹患首发癌后一般会接受更为正规的定期复查，可能导致次发癌能在更早期的时候被发现，这可能也是本研究中早期患者占绝大多数的原因之一。

DPLC预后较肺内转移好，对于影像学高度怀疑DPLC的患者，如心肺功能耐受，建议积极手术治疗。如无淋巴结及远处转移，尽可能行根治性治疗，较小的病灶行肺段切除术，原位癌可行局部切除术^[23]^。本研究60个病灶中大部分（55/60, 83.3%）都接受了手术治疗，中位随访时间70.5个月，全组患者中位OS 96.5个月（21个月-325个月），5年生存率86.7%，高于Takamochi等^[24]^报道的77.3%与Yang等^[25]^报道的75%，可能与近年来愈发积极的早期筛查和治疗相关。有研究认为同病理类型的DPLC预后优于不同病理类型^[26, 27]^，但亦有*meta*分析认为同种病理类型预后较好的原因是其包含了过多的原位或微浸润腺癌影响了生存分析的结果^[14]^，而本研究显示病理类型是否相同对DPLC预后无影响。Aziz等^[28]^的研究显示第二与第一原发癌时间间隔越长预后越好，并归因于间隔时间越长第二原发癌的侵袭性越低，但在本研究中间隔时间对OS的影响并没有统计学意义。多数研究认为病灶位于同侧或对侧对DPLC预后的影响无统计学意义^[29, 30]^，近期的*meta*分析也得出肿瘤位于对侧或同侧对患者预后无影响^[31]^，本研究得出了同样的结论。mDPLC的诊断要求两次肿瘤间间隔不少于6个月，且在组织学类型相同时，无瘤生存期≥2年亦是诊断的标准之一，这似乎提示我们mDPLC患者的生存时间可能长于sDPLC患者，遗憾的是，本研究中并没有观察到这种现象（*P*=0.436），这也与先前李营等^[3]^的研究结果（*P*=0.620）一致，本研究中仅有3例sDPLC，阴性的结果可能与此有关。

目前公认的最有效的MPLC预后预测因子是淋巴结转移，本研究也显示淋巴结转移是DPLC的独立预后因素，淋巴结受累对于预后的影响不仅是因为淋巴结转移一定意义上代表着疾病的进展和侵袭，也可能是因为淋巴结转移与DPLC来源于相同克隆（same clonality）相关，而同克隆来源对于DPLC来说往往预示着较差的预后^[32]^。多数研究发现，最高TNM分期相对于淋巴结转移来说不能很好的预测DPLC预后^[33-36]^，本研究中最高TNM分期对于DPLC的OS亦不具有统计学意义的影响，可能是最高TNM分期本身对于DPLC的预后价值较小，也可能是受限于本研究较小的样本量，我们期待有大规模的临床研究来证实对于DPLC是否有必要建立新的预后评价体系。烟草对于DPLC的影响亦需受到重视，多项研究指出双原发癌的发生与吸烟相关^[37-39]^，而初次诊断肺癌后两年内戒烟的患者发生mDPLC的风险明显降低^[40]^，本研究中重度吸烟的患者比例高达50.0%，并且发现重度吸烟史是DPLC的独立预后因素，这显示烟草不仅参与了DPLC的发生，也与其发展关系密切。烟草对于DPLC的影响一方面可能与烟草暴露导致患者基因（如*p53*等）改变有关^[41]^，也与气道长期接触致癌物导致的区域癌化相关^[42, 43]^。

虽然MPLC中80%左右均为DPLC^[2, 3]^，但本研究受限于较小的样本量，以及未能纳入多于两个病灶的MPLC患者，不能十分全面地反映MPLC的特性，我们期待更大样本以及包含更多分子基因标记物的研究来进一步完善MPLC的诊疗策略。

综上所述，DPLC的诊断需要多学科综合参与以提高准确性，确诊后应给予以积极手术为主的综合治疗，对于DPLC原发肺癌患者应执行更为严格的戒烟策略以期改善其预后。
